# Effect of Antithrombotic Therapy on Secondary Bleeding After Proctological Surgery

**DOI:** 10.7759/cureus.14983

**Published:** 2021-05-12

**Authors:** Ryo Maemoto, Shingo Tsujinaka, Yasuyuki Miyakura, Erika Machida, Taro Fukui, Nao Kakizawa, Sawako Tamaki, Hideki Ishikawa, Toshiki Rikiyama

**Affiliations:** 1 Surgery, Saitama Medical Center, Jichi Medical University, Saitama, JPN

**Keywords:** proctological surgery, antithrombotic therapy, antiplatelet drugs, anticoagulant drugs, secondary bleeding

## Abstract

Introduction

Surgery for patients taking antithrombotic drugs for the prevention and treatment of cardiovascular disease, including anticoagulants and antiplatelet drugs, is increasing because of the aging society. In patients with moderate to high risk for cardiovascular events, receiving antiplatelet therapy, and requiring noncardiac surgery continuing antiplatelet drugs perioperatively is recommended. To date, there have been limited reports on the risk of secondary bleeding after proctological surgery in patients who are administered antithrombotic drugs. The purpose of this study was to identify the incidence and severity of secondary bleeding after proctological surgery for patients with or without antithrombotic therapy.

Methods

We retrospectively identified 113 patients who underwent proctological surgery in our hospital from March 2009 to February 2019. In general, antiplatelet drugs were continued and anticoagulant drugs were either substituted or withdrawn prior to surgery. The severity of secondary bleeding was classified as mild, moderate, or severe.

Results

Eighteen patients underwent antithrombotic therapy (A group) and 95 patients did not undergo antithrombotic therapy (N group). Secondary bleeding was observed in nine patients (8.0%) and patients in the A group exhibited a significantly higher rate of secondary bleeding than those in the N group (39% vs. 2.4%, P < 0.01). The median interval from surgery to the onset of secondary bleeding was five days (range: 0-11). The severity of bleeding was the highest in patients administered direct oral anticoagulants (DOAC) and was the lowest in those administered aspirin. There was no mortality or cardiovascular event.

Conclusion

Antithrombotic therapy carries a high risk of secondary bleeding after proctological surgery. Delaying the postoperative resumption of anticoagulants is considered while balancing the risk of postoperative thromboembolic complications against secondary bleeding.

## Introduction

Surgery for patients taking antithrombotic drugs for the prevention and treatment of cardiovascular disease, including anticoagulants and antiplatelet drugs is increasing gradually because of the aging society. Discontinuation of antiplatelet drugs in the perioperative period could lead to a cardiovascular event in moderate to high-risk patients at a high rate [1-3]. Nowadays, in patients with moderate to high risk for cardiovascular events, such as after coronary artery stenting, receiving antiplatelet therapy, and requiring noncardiac surgery, continuing antiplatelet drugs perioperatively is recommended by recent clinical practice guidelines [4-5]. Furthermore, anticoagulation bridging therapy is recommended in patients with high risk for thromboembolism, including those with a mechanical heart valve, atrial fibrillation, and venous thromboembolism.

Several studies have evaluated the bleeding risk in patients who require noncardiac surgery with antithrombotic drugs [6-8]. Despite an increased risk of transfusion, continuing antithrombotic therapy during noncardiac surgery is feasible. To date, there have been limited reports on the risk of secondary bleeding after proctological surgery in patients who are administered antithrombotic drugs [9-11]. The purpose of this study was to identify the incidence and severity of secondary bleeding after proctological surgery in our hospital.

A preprint of this article was previously posted to Research Square on May 13, 2020 (https://www.researchsquare.com/article/rs-27989/v1).

## Materials and methods

This was a retrospective observational study conducted in a single institution and was approved by the Bioethics Committee for Clinical Research, Saitama Medical Center, Jichi Medical University (#S19-159). A total of 113 consecutive patients who underwent proctological surgery from March 2009 to February 2019 were included in the study. Antithrombotic therapy consists of antiplatelet therapy and anticoagulant therapy. As a general rule in our hospital, we continued antiplatelet drugs for any proctological surgery. For patients receiving clopidogrel or cilostazol, we replaced their medications with aspirin three to 14 days prior to the surgery. If patients received doublet antiplatelet therapy, we replaced their medications with aspirin alone seven to 14 days prior to the surgery. Patients on anticoagulant therapy were either bridged to low-molecular-weight heparin (LMWH) if they had a high risk for thrombosis or withdrawn before surgery. For example, warfarin therapy and direct oral anticoagulants (DOAC) were withdrawn five days and one to two days prior to the surgery, respectively. About 12-24 hours after the surgery, anticoagulant therapy was resumed if there were no signs of bleeding.

We compared short-term perioperative results (such as intraoperative bleeding, postoperative bleeding, and postoperative hospital stays) between patients who either (A group) or not received antithrombotic therapy (N group). According to Piot et al. [9], the secondary bleeding was graded as minor if the patients were not readmitted (no hemodynamic disturbance and/or no serious underlying medical condition), moderate if the patients were readmitted (frequent heavy bleeding, severe associated pathology, hemodynamic variations, anxiety, and/or isolated patient), or severe if surgical hemostasis or blood transfusion was needed.

We used t-tests to compare the average values of continuous variables under normal distribution and Mann-Whitney U-tests to compare the median values of continuous variables under non-normal distribution. Fisher’s exact tests were used to compare the categorical variables between the groups. The threshold for significance was set at P < 0.05. All statistical analyses were conducted using EZR (Easy R; The R Foundation for Statistical Computing, Vienna, Austria) [12].

## Results

Patients’ demographic data are shown in Table 1, and the most frequent comorbidity was cardiovascular disease, which was observed in 39 (35%) patients.

**Table 1:** Patients’ demographic data BMI: body mass index; ASA-PS: American Society of Anesthesiologists Physical Status classification

Variables	Mean ± SD, Median (range) or N (%)
Age	57 ± 18
Male/Female	85/28
BMI	23.4 (15.8-63.7)
ASA-PS 1/2/3	27/58/28
Comorbidities	
Cardiovascular disease	39 (35)
Endocrine and metabolism disease	21 (19)
Inflammatory bowel disease	14 (12)
Gastrointestinal disease	9 (8.0)
Renal disease	8 (7.1)
Hematological disease	7 (6.2)
Infectious disease	5 (4.4)
Psychiatry and neurology disease	5 (4.4)
Respiratory disease	4 (3.5)
Immunological disease	2 (1.8)
Antithrombotic therapies	18 (16)
Reasons for antithrombotic therapies	
Atrial fibrillation	7
Ischemic heart disease	3
Valve replacement	3
Brain stroke	2
Coronary artery bypass graft	1
Hypoplastic left heart syndrome	1
Hemodialysis	1

Table 2 shows perioperative data, including surgical procedures, rate of antithrombotic therapies, and complications. Eighteen (15.9%) patients received antithrombotic therapy (A group) for primary and secondary prophylaxes. Seventeen (15.0%) patients exhibited complications, including secondary bleeding events in nine (8.0%) patients. The severity of these bleeding complications was minor in five, moderate in three, and severe in one patient. The secondary bleeding events were treated via ligation and excision in seven patients and simply via laying open in two patients.

**Table 2:** Perioperative data PPH: procedure for prolapse and hemorrhoids; DOAC: direct oral anticoagulants

Variables	Median (range) or N (%)
Hemorrhoid/Fistula/Fissure	52/56/5
Surgical procedures	
Ligation and excision	48 (43)
Ligation	2 (1.8)
PPH	2 (1.8)
Lay open	32 (28)
Seton	18 (16)
Coring out	6 (5.3)
Fissurectomy	5 (4.4)
Drugs for antithrombotic therapies	
Aspirin	5 (4.4)
Warfarin	10 (8.8)
DOAC	3 (2.7)
Complications	
Secondary bleeding	9 (8.0)
Infection	3 (2.7)
Recurrence	2 (1.8)
Others	3 (2.7)
The interval days from surgery to secondary bleeding	5 (0-11)
Intervention for secondary bleeding	
Trans anal hemostasis	4
Observation	4
Intravenous drip of hemostyptic	1

Table 3 shows comparison demographic and perioperative data between A group (n = 18) and N group (n = 95). Patients in the A group had a significantly higher age (68 ± 18 vs. 54 ± 17 years, P < 0.01) and a higher secondary bleeding rate (39% vs. 2.4%, P<0.01) than the N group. Sex, body mass index, American Society of Anesthesiologists Physical Status classification (ASA-PS), operative time, intraoperative bleeding, and postoperative length of stay did not differ between the A group and N group. The median interval from the completion of surgery to the onset of secondary bleeding was five days (range: 0-11 days).

**Table 3:** Comparison between the A group and N group Numbers are displayed by either mean ± standard deviation, median (range), or N (%) BMI: body mass index; ASA-PS: American Society of Anesthesiologists Physical Status classification

Variables	A group (n=18)	N group (n=95)	P-value
Age	68 ± 18	54 ± 17	<0.01
Male/Female	14/4	71/24	1
BMI (kg/m^2^)	22.4 (15.8-30.7)	23.5 (16.1-63.7)	0.16
ASA-PS 1/2/3	0/10/8	27/48/20	0.07
Operative time (min)	39 (22-98)	37 (9-140)	0.79
Intraoperative bleeding (mL)	5 (5-106)	5 (5-150)	0.72
Postoperative length of stay	5 (3-11)	4 (1-78)	0.63
Secondary bleeding	7 (39)	2 (2.4)	<0.01

Antithrombotic therapies and secondary bleeding events are described in Table 4. Patients receiving anticoagulant therapy (such as taking warfarin and DOAC) were more likely to experience more severe secondary bleeding complications as compared with those receiving antiplatelet therapy. There were no cardiovascular, pulmonary, or cerebral events or mortality.

**Table 4:** Antithrombotic therapies and secondary bleeding Severity of secondary bleeding based upon the Pigot et al. classification

Severity of secondary bleeding	Aspirin (n=5)	Warfarin and heparin-bridging (n=10)	DOAC (n=3)
Minor	2	1	1
Moderate	0	2	0
Severe	0	0	1

## Discussion

This study investigated the risk of secondary bleeding after proctological surgery in patients who received antithrombotic therapy. The results showed that patients with antithrombotic therapy exhibited a higher risk of secondary bleeding than the control group. According to previous studies, the frequency of secondary bleeding after hemorrhoid surgery is 0.6%-2.4% [13-15] and it is likely to occur on the sixth to ninth postoperative day [15-16]. In our report, the incidence of secondary bleeding was slightly higher because our hospital is one of the tertiary referral centers that treat high-risk patients. As a result, 25% of the patients enrolled in this study were ASA-PS3 and about 35% of patients had cardiovascular disease.

A previous study showed that perioperative antiplatelet therapy for noncardiac surgery confers minimal bleeding risk with no thrombotic complications, which indicated that antiplatelet therapy was safe perioperatively [6]. This was in agreement with the results of the current study. Previously, there has been a study on the risk of secondary bleeding in patients receiving perioperative antithrombotic therapy during proctological surgery [9]. The multivariate analysis in that study revealed that the administration of clopidogrel, discontinuing anticoagulant drugs, and bridging to LMWH were significantly high-risk factors of secondary bleeding. Our study also showed that anticoagulant therapy, including warfarin and DOAC, was associated with increased severity of secondary bleeding.

In this study, we showed that antithrombotic therapy during proctological surgery could lead to a high risk of secondary bleeding. Hence, additional postoperative care is required for such cases. Since there was no cardiovascular, pulmonary, or cerebrovascular event and mortality during the perioperative period, it was hypothesized that temporary modification or discontinuation of their medication could be done during proctological surgery for patients receiving antithrombotic therapy.

This study revealed that patients receiving perioperative antithrombotic therapy had a high risk for secondary bleeding after proctological surgery and the severity of secondary bleeding was the highest in patients receiving DOAC and the lowest in those receiving aspirin. Since secondary bleeding caused by aspirin tends to be mild, aspirin could be administered during proctological surgery while considering the risk of a cardiovascular event caused by withdrawal. The medications of the patients receiving anticoagulant therapy should be tailored, while considering the risk of cardiovascular events, to minimize the severity of secondary bleeding. Based on the risk of cardiovascular events associated with a mechanical heart valve, atrial fibrillation, and deep vein thrombosis, it is necessary to determine whether anticoagulant therapy can be discontinued temporarily or bridged to heparin before surgery. For instance, the CHADS-2 score (congestive heart failure, hypertension, age ≥75 years, diabetes mellitus, stroke [doubled]) and the CHA2DS2‐VASc score (congestive heart failure, hypertension, age > 75 [doubled], diabetes mellitus, previous stroke/TIA/TE [doubled], vascular disease, age 65-74 years, sex category [female])" are available for the thrombotic risk calculation of patients with atrial fibrillation [4,17]. It has previously been recommended that anticoagulant therapy should be resumed after 12-24 hours postoperatively (evening of the day of surgery or the next morning after surgery) [4], however, in this study, the patients received warfarin. Generally, with warfarin therapy, the mean time attaining an international normalized ratio (INR) ≧ 2.0 is about five days but the same effect is achieved in a shorter time with DOAC. Our results that the median onset of secondary bleeding was five days after surgery indicated that the resumption of DOAC could be delayed until the fifth postoperative day, which was more delayed than our current practice (after 12-24 hours after surgery). Although surgeons must have already acknowledged paying attention to patients with antithrombotic therapy when they undergo proctological surgery, the actual data on postoperative outcomes have been rarely available in the literature, particularly for those with DOAC. The incidence of postoperative bleeding and the timing of the discontinuation and resumption of antithrombotic drugs remains to be investigated, particularly for patients with DOAC. Previous review articles recommended the discontinuation of antiplatelet or anticoagulant therapy about five to seven days before and after any forms of surgery for hemorrhoids [18-19]. Yano et al. [15] reported that 23 out of 1294 patients (1.7%) underwent a second operation for postoperative bleeding after hemorrhoidectomy. In their study, 3.6% of the patients had previous use of anticoagulants and that did not correlate with the increased incidence of postoperative bleeding. They also described that anticoagulant therapy was routinely discontinued about three to 10 days before surgery and resumed seven days after surgery [15].

There were several limitations of this study. First, this study was performed retrospectively at a single institute and the number of cases was small. Second, our study employed both antiplatelet and anticoagulant therapies, which could lead to varied results in the target population due to the different purposes of each type of antithrombotic therapy.

## Conclusions

Antithrombotic therapy is associated with a high risk of secondary bleeding after proctological surgery, particularly in patients with anticoagulant therapy. Delayed postoperative resumption of anticoagulants may be considered in balancing the individual risk of postoperative thromboembolic complications against the risk of secondary bleeding. Future prospective studies with a larger number of patients are needed to determine the appropriate timing of resuming antithrombotic therapy.
